# Transcriptome analysis of *Clavibacter michiganensis* subsp. *michiganensis*-infected tomatoes: a role of salicylic acid in the host response

**DOI:** 10.1186/s12870-021-03251-8

**Published:** 2021-10-19

**Authors:** Naoki Yokotani, Yoshinori Hasegawa, Masaru Sato, Hideki Hirakawa, Yusuke Kouzai, Yoko Nishizawa, Eiji Yamamoto, Yoshiki Naito, Sachiko Isobe

**Affiliations:** 1 Kazusa DNA Research Institute, 2-6-7 Kazusa-Kamatari, Kisarazu, Chiba, 292-0818 Japan; 2 Institute of Agrobiological Sciences, National Agriculture and Food Research Organization, 2-1-2 Kannondai, Tsukuba, Ibaraki, 305-8602 Japan; 3 Bioproductivity Informatics Research Team, RIKEN Center for Sustainable Resource Science, 1-7-22 Suehiro-cho, Tsurumi, Yokohama, 230-0045 Japan

**Keywords:** *Clavibacter michiganensis* subsp. *michiganensis*, Plant immunity, RNA-seq, Salicylic acid, Tomato, Transcriptome

## Abstract

Bacterial canker of tomato (*Solanum lycopersicon*) caused by the Gram-positive bacterium *Clavibacter michiganensis* subsp. *michiganensis* (*Cmm*) is an economically important disease*.* To understand the host defense response to *Cmm* infection, transcriptome sequences in tomato cotyledons were analyzed by RNA-seq. Overall, 1788 and 540 genes were upregulated and downregulated upon infection, respectively. Gene Ontology enrichment analysis revealed that genes involved in the defense response, phosphorylation, and hormone signaling were over-represented by the infection. Induced expression of defense-associated genes suggested that the tomato response to *Cmm* showed similarities to common plant disease responses. After infection, many resistance gene analogs (RGAs) were transcriptionally upregulated, including the expressions of some receptor-like kinases (RLKs) involved in pattern-triggered immunity. The expressions of *WRKYs*, *NACs*, *HSFs*, and *CBP60s* encoding transcription factors (TFs) reported to regulate defense-associated genes were induced after infection with *Cmm*. Tomato genes orthologous to Arabidopsis *EDS1*, *EDS5/SID1*, and *PAD4/EDS9*, which are causal genes of salicylic acid (SA)-deficient mutants, were upregulated after infection with *Cmm*. Furthermore, *Cmm* infection drastically stimulated SA accumulation in tomato cotyledons. Genes involved in the phenylalanine ammonia lyase pathway were upregulated, whereas metabolic enzyme gene expression in the isochorismate synthase pathway remained unchanged. Exogenously applied SA suppressed bacterial growth and induced the expression of *WRKYs*, suggesting that some *Cmm*-responsive genes are regulated by SA signaling, and SA signaling activation should improve tomato immunity against *Cmm*.

**Supplementary Information:**

The online version contains supplementary material available at 10.1186/s12870-021-03251-8.

## Background

Bacterial canker is one of the most destructive diseases of tomato (*Solanum lycopersicon*) and is caused by the Gram-positive bacterium *Clavibacter michiganensis* subsp. *michiganensis* (*Cmm*) [1]. This disease is spread by seed transmission and impairs fruit yield due to leaf withering, stem canker, and bird’s eye spots on fruit [2]. Since there are no resistance cultivars and limited pesticides that are highly effective against *Cmm*, disease control of bacterial canker is limited to the maintaining disease-free seed and plant residues to prevent the spread of the bacteria [3–5]. Therefore, it is important to understand the mechanism of the host response to *Cmm* for the control of bacterial canker.

Plants have an immunity system through which they recognize the attack of pathogens and exert defense responses. Pattern-triggered immunity (PTI) is induced by recognizing structurally conserved molecules called microbe- or pathogen-associated molecular patterns (MAMPs/PAMPs) and damage-associated molecular patterns (DAMPs) through the pattern recognition receptors [6–8]. Receptor-like kinases (RLKs) play an important role in PTI against various pathogens in plants [6, 9]. MAMPs/PAMPs are molecules derived from pathogen, and one of them is peptidoglycan, accounts for the majority of the dry weight of Gram-positive bacteria; thus, it might be an important component in the interaction between tomato and *Cmm*. In Arabidopsis and rice, recognition of the glycan molecules that contain *N*-acetylglucosamine, including peptidoglycan from bacterial pathogens, is mediated by LysM receptor-kinase CERK1 [10, 11]. In tomato, *SlLYK1/Bti9* and *SlLYK13*, which are candidates for the orthologue of Arabidopsis and rice *CERK1s*, are involved in the PTI against the bacterial disease *Pseudomonas syringae* pv. *tomato* [12]. RLKs are also involved in recognition of DAMPs, molecules derived from damaged plant tissues inducing danger signal [8]. For example, Arabidopsis LecRK-1.8 recognize eNAD^+^ and positively regulate immunity to bacterial disease [13]. FER is reported to perceive rapid alkalinization factors and negatively regulate immunity [14]. When receptors in hosts recognize the MAMPs/PAMPs or DAMPs, immune signals are transmitted to the nucleus and lead to the transcriptional activation of defense-associated genes via various types of transcription factors (TFs), such as WRKYs, ERFs, NACs, and CBP60s [15–17]. Defense-associated genes encode proteins predicted to play roles in antimicrobial defense, mechanical protection, or the regulation of the hypersensitive reaction to resist pathogens [6, 18]. Genes commonly induced by a wide range of pathogens in various plant species include pathogenesis-related (PR) genes, which are often used as molecular markers of the defense response [18].

Plants also have another immune system called effector-triggered immunity (ETI). In this system, the disease response is triggered by the recognition of pathogen-derived effectors by the resistance (*R*) gene product, and the rapid and strong expression of defense-associated genes completely suppresses the growth of pathogens [7]. The products of the *R* genes are classified into several gene families based on the motifs of their encoding proteins, such as nucleotide-binding sites (NBSs), receptor-like proteins (RLPs), transmembrane coiled-coils (TM-CCs), and RLKs [19]. The genes encoding these proteins are called resistance gene analogs (RGAs), and there are more than 800 RGAs in the tomato genome [19–21]. The functions of RGAs are not limited to ETI and, in particular, some RLKs play roles for PTI and other biological aspects, such as growth, development, and the abiotic stress response [7, 9]. However, since the *R* gene against *Cmm* has not yet been discovered, there is currently no evidence that ETI is involved in the response to this disease in tomato.

In plant immunity, plant hormones, such as ethylene (ET), jasmonate (JA), and salicylic acid (SA), are often produced during infection and play important roles, such as in the transmission of immune signals to distant tissues as well as in the amplification, maintenance, and suppression of the signals [22, 23]. ET is formed from S-adenosyl-l-methionine via a two-step reaction of conversion to 1-aminocyclopropane-1-carboxylate (ACC) by ACC synthase (ACS) and subsequent conversion to ET by ACC oxidase (ACO) [24]. JA is synthesized from linolenic acid by LOX, AOS, AOC, and OPR3 and is subsequently converted to the active form, JA-Ile, by JAR1 [25]. In plants, SA may be formed through one of two pathways [26–28]. One pathway is the isochorismic acid by isochorismate synthase (ICS) pathway, which is a three-step reaction of conversion from chorismic acid to isochorismic acid by ICS (ICS1/SID2/EDS16), conversion to isochorismoyl-glutamate by isochorismoyl-glutamate synthase (PBS3/IGS), and conversion to SA by pyruvoyl-glutamate lyase (EPS1/IPGL) [29, 30]. The other is the phenylalanine ammonia lyase (PAL) pathway, where SA is synthesized from L-phenylalanine via *trans*-cinnamic acid and benzoic acid. In the PAL pathway, conversions of L-phenylalanine to *trans*-cinnamic acid, *trans*-cinnamic acid to Cinnamoyl-CoA, and Cinnamoyl-CoA to benzoic acid are catalyzed by PAL, 4-coumarate:CoA ligase (4CL), and 3-hydroxyacyl-CoA dehydrogenase (AIM1), respectively [26–28]. In response to fungal disease, JA/ET and SA regulate resistance to necrotrophs and biotrophs, respectively, and they partially interact in an antagonistic manner in Arabidopsis [22, 31]. ET production is induced by infection with *Cmm* [32]. However, ET only regulates leaf blight symptoms and does not affect bacterial growth [32]. Application of benzothiadiazole (BTH), a functional analog of SA, induces resistance to *Cmm* [33]. On the other hand, *Pseudozyma aphidis*-induced resistance to *Cmm* in tomato is independent of SA [34].

To understand plant immunity, analyzing the host transcriptome is a useful approach. To date, a number of studies have investigated changes in the transcriptome after infection with *Cmm*. For example, microarray analysis reported that infection with *Cmm* to compatible tomato cultivar induces expression of various genes associated with redox regulation, protein turnover, and ethylene biosynthesis [32]. Studies of proteomic analysis have reportedly identified the *Cmm*-responsive proteins, including PR proteins and antioxidant enzymes [35, 36]. Comparison of transcriptome between resistant and cultivated tomato by RNA-seq analysis revealed that various genes involved in defense and stress response are upregulated in resistant line [37]. However, there is little knowledge on the signaling proteins and plant hormones that regulate PTI to *Cmm*. The aim of this study was to analyze the host defense response by utilizing whole genome transcriptome and modern gene annotation at the molecular level to control *Cmm* effectively. In this study, we analyzed the transcriptome sequence in response to infection over time using RNA-seq analysis. We identified a relationship between bacterial growth, disease symptoms, and the expression of defense-related genes. We also revealed a role of SA in the host response to *Cmm*.

## Results

### Colonization of *Cmm* and symptoms developed in tomato cotyledons

**Fig. 1:**
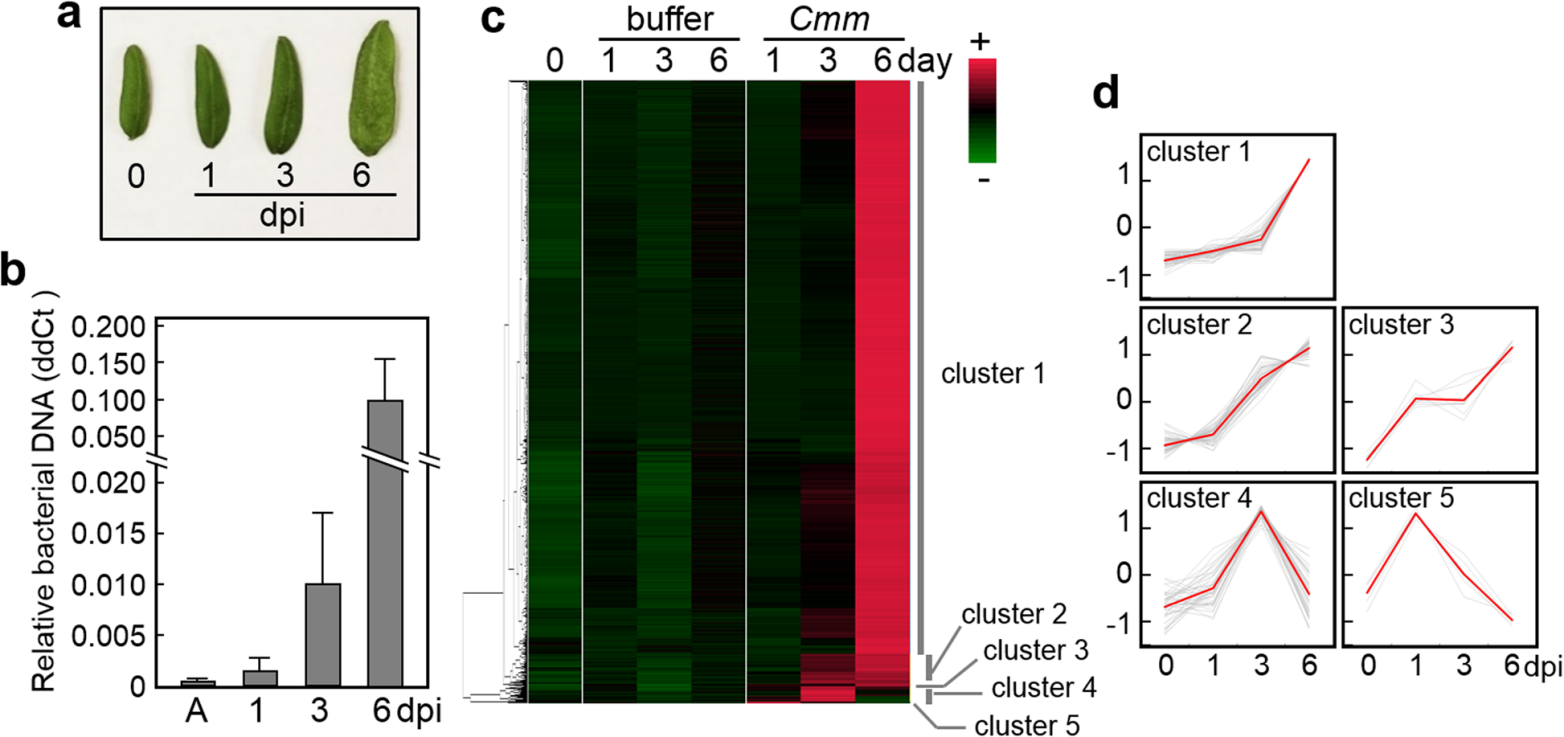
Expression patterns of genes upregulated in tomato cotyledon after infection with *Cmm* (upregulated differentially expressed genes (DEGs)). **a** Disease symptom of the tomato cotyledons after infection with *Cmm*. The values represent the number of days after inoculation. **b** Quantification of the bacterial biomass by measuring the amount of genomic DNA in tomato cotyledons. The amount of *Cmm ptssk* DNA relative to tomato *COX* DNA was determined by qPCR analysis. *A* represents cotyledons immediately after inoculation. Values are represented as means ± standard deviation for six seedlings. **c** Cluster analysis of upregulated DEGs; 0 dpi refers to uninoculated cotyledons. The phylogenetic tree was constructed by the unweighted pair group method with arithmetic mean analysis. Z-score-transformed relative gene expressions were visualized with a heatmap. Genes were divided into the following five clusters: low expression until 3 dpi then greatly increasing at 6 dpi (cluster 1); increased expression at 3 dpi then maintaining a high level until 6 dpi (cluster 2); increased expression at 1 dpi then maintaining a high level until 6 dpi (cluster 3), increased expression until 3 dpi then deceasing at 6 dpi (cluster 4), and increased expression at 1 dpi then decreasing at 3 dpi (cluster 5). **d** Time-series expression levels of genes in each cluster. Z-scored expression data are shown as gray lines. In clusters 1 and 2, the data of 50 genes were randomly selected and are shown. The average of each expression level is shown as a red line

No symptoms were detected within 1 day post inoculation (dpi). At 3 dpi, small yellow spots and bumpy surfaces were detected. At 6 dpi, severe disease symptoms, including chlorosis and imbibition, were observed all over the cotyledons (Fig. 1a). The bacterial biomass in the cotyledons after infection was measured by quantitative polymerase chain reaction (qPCR) analysis which is a simple and accurate method for bacterial quantification (Fig. 1b). Because the amount of bacteria inoculated was small, *Cmm* DNA was detected at low levels from the cotyledons immediately after inoculation. At 1 dpi, *Cmm* DNA was detected from all samples tested despite no obvious symptoms. From 3 to 6 dpi, the bacterial biomass increased dramatically with the spread of disease symptoms. In order to analyze the changes in the host transcriptome over time during infection, we established a method of infiltrating cotyledons that allows stable observation of bacterial growth and development of disease symptoms in a short period of time. Sampling was performed at three points: 1 dpi, where bacterial growth was detected but no disease symptoms were observed; 3 dpi, where minor disease symptoms were observed; and 6 dpi, where severe disease symptoms were observed.

### Transcriptome profiling of tomato in response to infection by *Cmm*

The analysis generated 21.8 to 28.5 million raw reads for each sample, and 97.3 to 98.5% of the obtained reads were properly mapped to the *S. lycopersicon* reference genome (SL4.0) and International Tomato Annotation Group *S. lycopersicon* gene annotation (ITAG4.0) (https://solgenomics.net/organism/Solanum_lycopersicum/genome), which contained 34,075 annotated genes (Table S1). The mean TPM values and the correlation coefficient values in samples were shown in Table S2. In this study, 9087 genes were identified as differentially expressed genes (DEGs) based on the following selection: a maximum mean transcripts per million (TPM) value of seven groups > 10, a false discovery rate (FDR) < 0.01, and the mean max/min difference being > 2. Among them, 1788 genes were identified as upregulated DEGs based on a fold-change > 2 when comparing a maximum mean TPM value in 0 day and in three time points of buffer treatment conditions with those in the maximum mean TPM value in three time points of *Cmm* inoculation conditions respectively (Table S3). In the same way, 540 genes were identified as downregulated DEGs based on a fold-change < 0.5 when comparing a minimum mean TPM value in 0 day and in three time points of buffer treatment conditions with those in the minimum mean TPM value in three time points of *Cmm* inoculation conditions respectively. (Table S4).

The expression pattern of the upregulated DEGs was divided into five clusters by hierarchical clustering based on the time-series TPM values. Most of the upregulated DEGs (1646 genes) were specified as cluster 1, in which expression levels dramatically increased at 6 dpi (Fig. 1c, d). Among the remainder, 86, 8, 43, and 5 genes were assigned to cluster 2, 3, 4, and 5, respectively.

In the upregulated DEGs, 46 Gene Ontology (GO) terms were over-represented (Table S5). These included immune-related GO terms, such as defense response to fungus (GO:0050832), plant-type hypersensitive response (GO:0009626), response to biotic stimulus (GO:0009607), and regulation of systemic acquired resistance (GO:0010112). In addition, GO terms associated with signaling pathways, such as the hormone-mediated signaling pathway (GO:0009755), regulation of SA biosynthetic process (GO:0080142), calcium signaling (GO:0009931), redox regulation (GO:0006749), and protein phosphorylation (GO:0006468), were over-represented. Moreover, GO terms associated with the extracellular (GO:0005615) and cell surface receptor signaling pathway (GO:0007166) were over-represented. In the downregulated DEGs, 15 GO terms were over-represented (Table S5), which included GO terms associated with photosystems (GO:0015979), response to high light intensity (GO:0009644), and response to light stimulus (GO:0009416). In cotyledons after infection with *Cmm*, 166, 3793, and 1993 genes were increased more than 2-fold compared to buffer-treated cotyledons at 1, 3, and 6 dpi respectively (Table S6). In cotyledons after infection with *Cmm*, 136, 77, and 1097 genes were decreased less than 0.5-fold compared to buffer-treated cotyledons at 1, 3, and 6 dpi respectively (Table S6). GO enrichment analysis of each time point revealed that GO terms associated with defense response to fungus (GO:0050832) and hormone-mediated signaling pathway (GO:0009755) were over-represented at 6 dpi (Table S6).

### Expression of PR genes after infection with *Cmm* in tomato

**Fig. 2:**
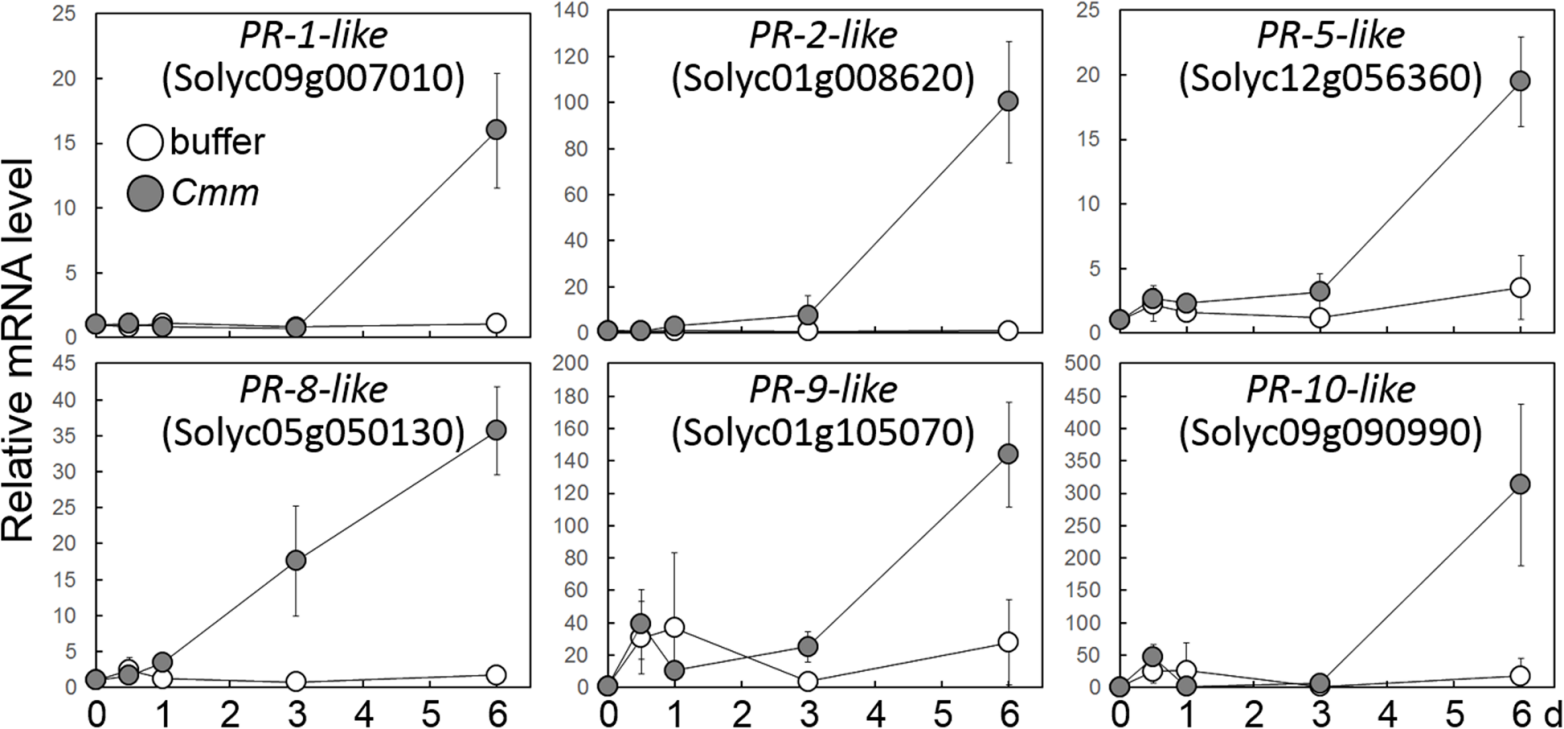
Expression of pathogenesis-related genes in tomato plants after infection with *Cmm*. The transcript levels in tomato cotyledons were quantified by qRT-PCR analysis and expressed relative to the transcript level at 0 day, which was assumed to be one. Data are represented as mean values ± the standard deviation for three replicates

After *Cmm* infection of tomato cotyledons, the expression of 40 PR genes belonging to six classes was induced (Table S3). The gene-set hypergeometric enrichment test demonstrated that the PR gene homologs were significantly (*p*-value < 0.05, hypergeometric distribution test) over-represented in the upregulated DEGs. As shown in Fig. 2, quantitative reverse transcription-PCR (qRT-PCR) analysis confirmed the expression data of PR genes by RNA-seq analysis. The data of qRT-PCR are consistent with that of RNA-seq analysis.

### Expression of the genes involved in defense signaling after infection with *Cmm* in tomato

After *Cmm* infection, 186 RGAs, including 24 NBSs, 25 RLPs, 15 TM-CCs, and 122 RLKs, were transcriptionally upregulated (Table 1). NBSs, RLPs, TM-CCs, and RLKs were significantly (*p*-value < 0.05, hypergeometric distribution test) over-represented in upregulated DEGs. Based on grouping by Sakamoto et al. (2012) [21], *Cmm*-responsive RLK genes were categorized into various groups, such as LRR, receptor-like cytosolic kinase (RLCK), and LysM. Upregulated DEGs included *SlSERK3s*, *TFT1/TARK1*, *SOBIR/EVR*, and *SlLYKs* [12, 38, 39]. Upregulated DEGs also included the RLCK gene *ACIK1* [40] and two *FER*-like genes; *FER4* and *FER10* [41].

**Table 1:** Genes encoding resistance gene analogs (RGAs) and transcription factors (TFs) upregulated after infection with *Cmm*

Family^a^	Group	Number	Gene symbol^b^
RLK	Total	122	
	DUF26	10	
	10 L2	8	
	LRR	38	SlSERK3A, SlSERK3B, TFT1/TARK1
	LysM	3	SlLYK1/Bti9, SlLYK4, SlLYK9
	SD1a	14	
	SD2b	10	
	WAK	2	
	WAK/LRK10L1	8	
	LEC	3	
	RLCK	20	ACIK1
	other	6	SOBIR1/EVR, FER4, FER10, LESK1
NBS		24	
RLP		25	
TM-CC		15	
WRKY	Total	22	
	Group I	3	SlWRKY4, SlWRKY31, SlWRKY33
	Group II-a	3	SlWRKY39, SlWRKY45, SlWRKY46
	Group II-b	3	SlWRKY6, SlWRKY16, SlWRKY17
	Group II-c	4	SlWRKY23, SlWRKY51, SlWRKY55, SlWRKY75
	Group II-d	1	SlWRKY8
	Group III	8	SlWRKY41, SlWRKY42, SlWRKY53, SlWRKY54, SlWRKY58, SlWRKY59, SlWRKY80, SlWRKY81
NAC	Total	14	
	Group I	4	SlNACMTF3, SlNACMTF12
	Group II	3	SlNACMTF8
	Group III	2	SlNAC1, SlNAC2
	Group VI	2	SlNACMTF11
	other	3	SlJUB
HSF		5	SlHsfA4b, SlHsfA4c, SlHsfB1, SlHsfB2b, SlHsfB3a
CBP60		5	

^a^Gene family significantly enriched in up-regulated DEG by hypergeometric distribution test (*p* < 0.05)

^b^Genes whose symbols have been reported

Overall, 90 TF genes comprising 24 families in PlantTFDB 5.0 [42] and CBP60s were upregulated after infection and 22 WRKYs, 14 NACs, 5 CBP60s, and 5 HSFs were significantly (*p*-value < 0.05, hypergeometric distribution test) over-represented (Table 1 and S7). The 22 *Cmm*-responsive WRKYs consisted of all six groups, Group I, II-a, II-b, II-c, II-d, and III [43, 44]. When applied to the phylogenetic classification of Jensen et al. (2010) [45], the 14 *Cmm*-responsive NAC genes were classified into the seven following groups: I, II, III, VI, V, IV, and IX. They included the four NAC genes encoding SlNACMTF3, 8, 11, and 12, which each have a membrane binding domain [46].

### Increase of SA levels and the expression of the SA-associated genes after infection with *Cmm* in tomato

**Fig. 3:**
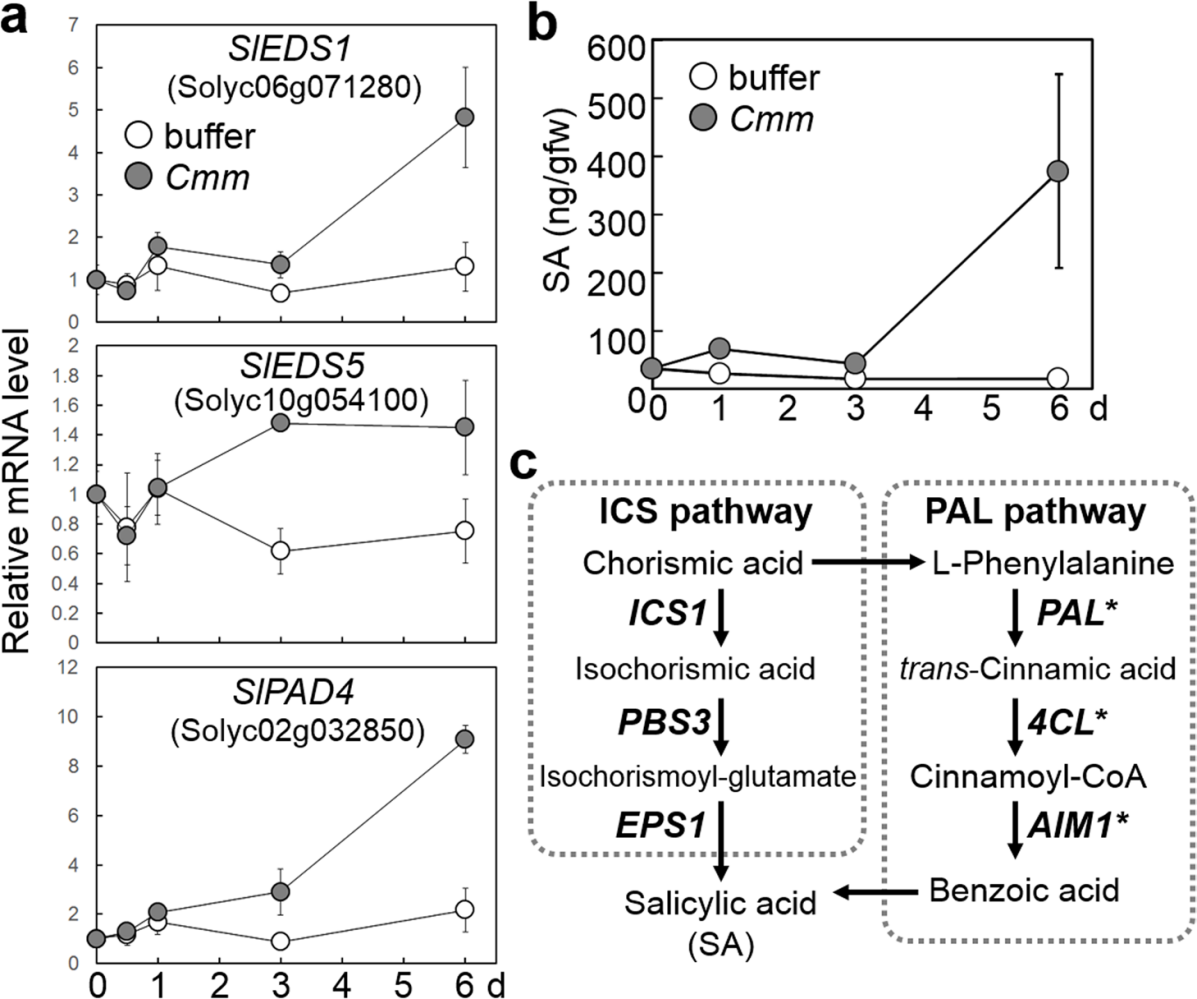
Accumulation of salicylic acid (SA) and the expressions of genes related to SA accumulation in tomato plants after infection with *Cmm*. **a** The expression of genes responsible for SA accumulation in tomato cotyledon after infection with *Cmm*. The transcript levels were quantified by quantitative reverse transcription-polymerase chain reaction and were expressed relative to the transcript level at 0 day, which was assumed to be one. Data are represented as mean values ± standard deviation for three replicates. **b** The level of SA accumulation in the tomato cotyledons after infection with *Cmm*. Data are represented as mean values ± standard deviation for six replicates. **c** Changes in the expression of genes involved in the possible SA biosynthetic pathway. Genes marked with an asterisk were upregulated in the tomato cotyledon after infection with *Cmm*. The upregulated genes identified by RNA-sequencing analysis (Table S3) were four phenylalanine ammonia lyase (*PAL*) genes (*SlPAL2/Solyc09g007900*, *SlPAL4/Solyc09g007920*, *SlPAL5/Solyc09g007910*, and *SlPAL6/Solyc05g056170*), three 4-coumarate:CoA ligase homologs (*Sl4CL/Solyc03g117870*, *Solyc06g068650*, and *Solyc12g042460*), and three 3-hydroxyacyl-CoA dehydrogenase homologs (*Solyc07g019670*, *Solyc12g007170*, and *Solyc08g068390*)

As described above, SA-associated GO terms, including regulation of SA biosynthetic process (GO:0080142) and regulation of systemic acquired resistance (GO:0010112), were over-represented in the upregulated DEGs (Table S5). The expressions of *Solyc06g071280*, *Solyc10g054100*, and *Solyc02g032850*, which are tomato orthologous genes for Arabidopsis *EDS1* [47], *EDS5/SID1* [48], and *PAD4/EDS9*, [49] respectively, were induced after infection (Table S3). We named these three genes *SlEDS1*, *SlEDS5*, and *SlPAD4*, respectively, and their expression after infection with *Cmm* was validated by qRT-PCR (Fig. 3a).

As shown in Fig. 3b, SA levels in tomato cotyledons were lower than 40 ng/gfw under normal conditions and were not changed by buffer treatment. *Cmm* infection increased SA levels in cotyledons within 1 dpi. SA levels showed a similar pattern to the expression of defense-associated genes, increasing substantially on 6 dpi to approximately 370 ng/gfw. The SA levels in *Cmm*-inoculated cotyledons were significantly higher than those in the buffer-treated cotyledons at 1, 3, and 6 dpi (*p*-value < 0.005, t-test). On the other hand, the levels of JA in the cotyledons were below the detection limit regardless of the presence or absence of *Cmm* infection (data not shown).

We attempted to identify candidate genes involved in the regulation of SA levels from upregulated DEGs. In the PAL pathway, one of the candidate SA synthesis pathways, four PAL genes (*SlPAL2/Solyc09g007900*, *SlPAL4/Solyc09g007920*, *SlPAL5/Solyc09g007910*, and *SlPAL6/Solyc05g056170*), three 4CL homologs (*Sl4CL/Solyc03g117870*, *Solyc06g068650*, and *Solyc12g042460*), and three AIM1 homologs (*Solyc07g019670*, *Solyc12g007170*, and *Solyc08g068390*) were identified in the upregulated DEGs (Fig. 3c and Table S3). Whereas there were no enzyme genes of the ICS pathway in the upregulated DEGs (Table S3).

### Effect of SA on the colonization of *Cmm* and defense-associated genes in tomato

**Fig. 4:**
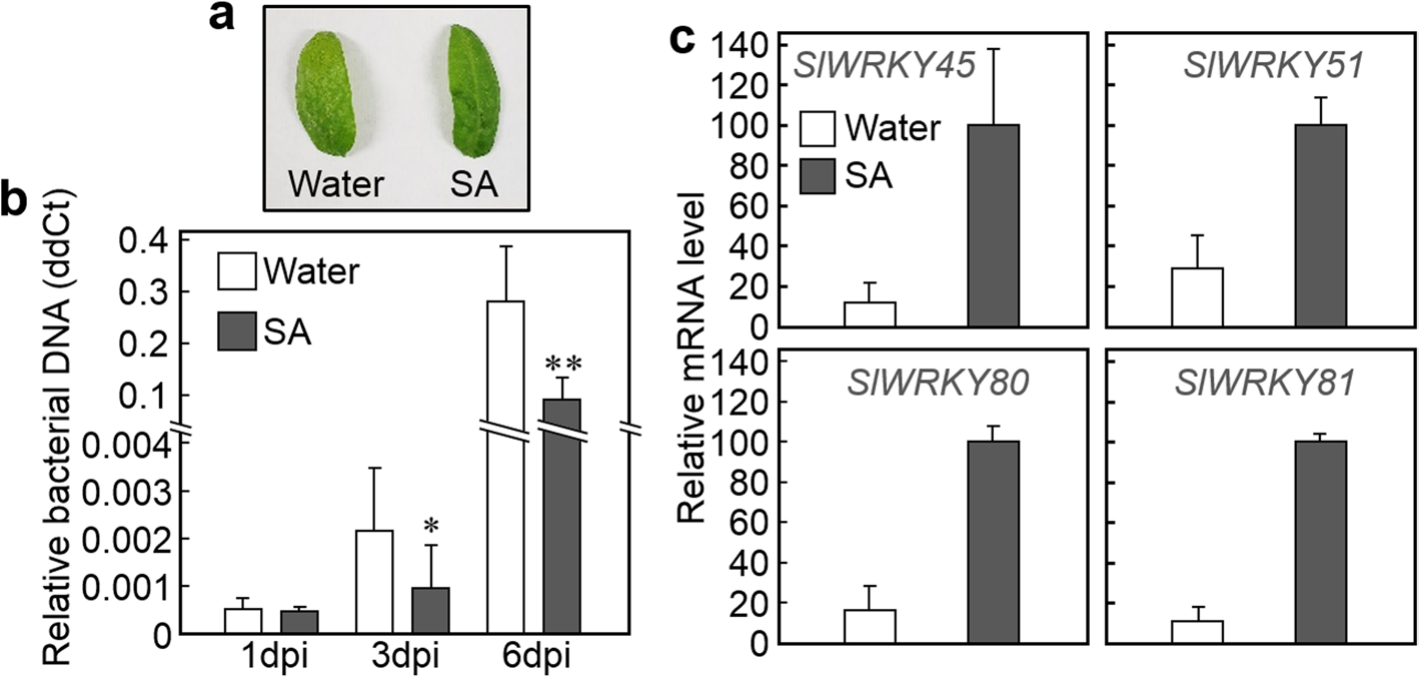
The effect of salicylic acid (SA) on the growth of *Cmm* and expression of WRKY genes in tomato plant. **a** Disease symptom of the SA-treated tomato cotyledons at 6 dpi. **b** The effect of exogenous applied SA on the growth of *Cmm* in tomato cotyledons. Tomato seedlings were transplanted to soil with or without 1 mM of SA 24 h before inoculation. The amount of *Cmm ptssk* DNA relative to tomato *COX* DNA was determined by quantitative polymerase chain reaction analysis. Values are represented as the means ± standard deviation for six seedlings. Significantly lower values compared with water control are denoted by asterisk (*p-value < 0.05; **p-value < 0.01, t-test). **c** The effect of SA on the expression of WRKY genes in tomato cotyledons. Tomato seedlings were transplanted to soil with or without 1 mM of SA and incubated for 6 h. Transcript levels were quantified by quantitative reverse transcription-polymerase chain reaction and expressed relative to the transcript level in SA-treated cotyledons, which was assumed to be 100. Data are represented as the mean values ± standard deviation for three replicates

Disease symptoms in SA-treated cotyledons at 6 dpi were less severe than those of cotyledons without SA (Fig. 4a). qRT-PCR analysis revealed that the bacterial biomass in SA-treated cotyledons was significantly (*p*-value < 0.05, t-test) smaller than that in non-treated cotyledons (Fig. 4b). To examine the effect of SA on the immunity of tomato plants, qRT-PCR analysis was conducted to determine the expression of disease-associated genes of upregulated DEGs. Interestingly, the expressions of four WRKY genes; *SlWRKY45*, *SlWRKY51*, *SlWRKY80*, and *SlWRKY81*, were significantly (*p*-value < 0.01, t-test) upregulated by SA treatment (Fig. 4c). Expression of WRKY genes in tomato plants after infection with *Cmm* were tested by qRT-PCR (Fig. S1).

## Discussion

In this study, with the aim of understanding the defense response to infection by *Cmm*, the transcriptome sequences were analyzed over time by RNA-seq analysis in tomato cotyledons. In the cotyledons inoculated with *Cmm*, disease symptoms and bacterial growth were observed. This experimental system was used to analyze the tomato transcriptome sequences over time after *Cmm* infection. RNA-seq analysis demonstrated that the 1788 genes were upregulated DEGs in response to *Cmm* (Fig. 1 and Table S3). This indicated that approximately 7% of tomato genes responded to *Cmm* within 6 dpi under the experimental conditions. GO analysis demonstrated that the GO terms associated with plant defense response against pathogens, such as defense response to fungus (GO:0050832), plant-type hypersensitive response (GO:0009626), and response to biotic stress (GO:0009607), were over-represented in upregulated DEGs (Table S5). Moreover, the gene set over-represented the GO terms related to defense signal transduction, such as phosphorylation, calcium signaling, redox regulation, and plant hormone signaling, and these biological processes are crucial for signal transduction during PTI [6, 18, 50]. These results suggest that a full PTI process is activated within 6 days after *Cmm* inoculation. During the infection process, the host induced the expression of six classes of PR genes (Fig. 2). Previously, transcriptome and proteome analysis demonstrated that expressions of PR genes were induced after infection with *Cmm* [35–37]. Because some classes of PR proteins show antimicrobial activities against bacterial pathogens [18], this upregulation of the PR genes may contribute to suppressing the colonization of *Cmm* in tomato plants.

RNA-seq analysis also showed that 540 genes were downregulated DEGs, and GO analysis demonstrated that the genes functionally related to photosynthesis or light intensity response were over-represented in this gene set (Table S4, S5). The downregulation of photosynthesis-related activities in response to pathogen infection also occurs in other plants, such as Arabidopsis, tobacco, *Brachypodium distachyon*, and oak [51–54]. In the context of plant resource allocation from growth to defense, the downregulation of photosynthesis-related activities could be a part of plant immunity, and our RNA-seq analysis may illuminate a growth and immunity trade-off during the interaction between tomato and *Cmm* [55].

A considerable increase of bacterial growth with severe symptoms was observed in host plants at 6 dpi, and the host transcriptional change in response to the pathogen inoculation was much more pronounced at 6 dpi than those at 1 and 3 dpi (Fig. 1). This relatively slow timing of the transcriptional response could result from a compatible interaction between *Cmm* and tomato. In Arabidopsis, inoculation of an incompatible strain of the bacterial pathogen *P. syringae* pv. *tomato* induces a rapid transcriptional response, including defense-associated genes, whereas that of a compatible strain delays transcriptional reprogramming [56]. A similar host transcriptional response occurs in interactions between *B. distachyon* and the fungal pathogen *Rhizoctonia solani* [52]. A large amount of *Cmm* colonization may be necessary to induce substantial transcriptional changes in susceptible tomato cultivars. Severe disease symptoms were observed in the day 6 plants, suggesting that *Cmm*-responsive genes respond to DAMPs and stress. It is known that pathogens have mechanisms to suppress host defense responses. Because a successful pathogen produces and secretes effector proteins to disturb the host immune system [57], the host transcriptional change, including the induction of defense-associated genes, may be suppressed or delayed by effector proteins of *Cmm* at an initial infection stage.

Plants have a large number of genes for RGAs, but not much is known about genome-wide analyses of these roles in PTI. After *Cmm* infection, 188 RGAs, including 24 NBSs, 25 RLPs, 15 TM-CCs, and 122 RLKs, were transcriptionally upregulated (Table 1). Some *Cmm*-responsive RLKs have been reported to regulate PTI. LysM RLKs recognize the pathogen cell wall polysaccharide-derivative MAMPs, including chitin and peptidoglycan. Three LysM RLK genes; *SlLYK1/Bti9*, *SlLYK4*, and *SlLYK9*, are induced by *Cmm* infection and may be involved in the recognition of peptidoglycans, which are the bulk of the dry matter weight of Gram-positive bacteria [12]. In Arabidopsis, FER regulates rapid alkalinization factor -mediated inhibition of host immunity [14]. In tomato, FER-like RLKs are reported to regulate heat stress response [41]. *Cmm*-responsive FER4 and 10 might be involved in DAMPs-mediated stress signaling. RLKs carrying the LRR domain bind proteinaceous ligands [9]. Among *Cmm*-responsive LRR-RLK genes, *SlSERK3A, SlSERK3B,* and *TFT1/TARK1* regulate PTI against bacterial disease [38, 39]. These results suggest that unidentified proteinaceous MAMPs or DAMPs may related to PTI in the host plant. Thus, many RLKs involved in PTI respond to infectious diseases, suggesting that PTI is regulated at the transcriptional level. In the present study, the levels of many members of non-RLK RGAs, including those of NBSs, RLPs, and TM-CCs, increased and were over-represented in *Cmm*-infected cotyledons. It is unlikely that these genes are involved in ETI in the tomato cotyledons used in this study because of the compatible combination. NBSs may be involved in defense responses independent of ETI. Maize *ZmNBS25* responds to infection with *Bipolaris maydis* and induces disease resistance in rice and Arabidopsis upon overexpression [58]. In Arabidopsis, overexpression of ADR1 increases resistance to virulent strains of fungal pathogens [59]. Since pathogens are a compatible race, resistance is unlikely to be directly involved in ETI caused by effector–NBS interactions. Similarly, *Cmm*-responsive RGAs, including NBSs, RLPs, and TM-CCs, may be involved in the regulation of plant immunity by acting differently from ETI.

TFs that directly regulate the expression of defense-associated genes play an important role in plant immunity [6, 16]. In this study, the TFs WRKYs, NACs, HSFs, and CBP60s were over-represented in *Cmm*-responsive genes. Previous studies have demonstrated that infection with *Cmm* induces expression of ERF TF genes [32, 37, 60]. In the present study, five ERF genes were transcriptionally upregulated by *Cmm* but not over-represented because of the large population size (Table S7). In tomato, 81 WRKYs were previously identified and phylogenetically classified into the following six groups: I, II-a, II-b, II-c, II-d, and III [44]. WRKY is widely conserved in higher plants and is involved in the W-box-mediated expression of defense-associated genes [16, 43]. *Cmm*-responsive SlWRKY8 is a transcriptional activator that promotes the expression of PR genes and resistance to the bacterial pathogen *P. syringae* [61]. In contrast, SlWRKY45 suppresses root knot nematode resistance, and many other Group IIa transcriptional repressors negatively regulate disease resistance [62]. Members of Group I and Group III, a large number of which are induced by *Cmm* infection, may also be important for the disease response [63, 64]. Thus, a number of WRKY transcriptional activators and repressors should be involved in regulating the transcription of defense-associated genes both positively and negatively, respectively, after infection with *Cmm*. NACs are a large family of TFs involved in plant growth, development, hormone signaling, and biotic and abiotic stress response [15]. Overall, 14 *Cmm*-responsive NAC proteins were classified into seven groups using a method based on amino acid sequence homology [45]. Interestingly, four of the 13 membrane-bound NACs in the tomato genome were responsive to *Cmm*. SlNACMTF3 and SlNACMTF8 were induced by an infectious disease and may be involved in the defense response [46]. GO enrichment analysis suggested that genes associated with the plasma membrane and extracellular space play important roles in response to infection with *Cmm* (Table S5). Membrane-bound NAC TFs may play roles in mediating signal transduction from the extracellular environment to the nucleus. In this study, five HSF genes were also induced after infection with *Cmm*, implying that the *Cmm* response may be partly related to the abiotic stress response [65]. CBP60s are a family of calmodulin-binding domain-containing proteins that are TFs. In Arabidopsis, two CBP60 proteins; SARD1 and CBP60g, positively regulate immunity to bacterial disease via the expression of PR genes and SA synthesis genes [66, 67]. In this study, the tomato orthologue of *SARD1* (*Solyc12g036390* and *Solyc03g119250*) and *CBP60g* (*Solyc01g100240*) were induced by *Cmm* infection, suggesting that the functions of CBP60s in disease response are highly conserved in a wide range of plants.

In this study, the orthologues of the causative genes of the Arabidopsis disease-susceptible mutants; *eds1*, *eds5*, and *pad4*, which we named *SlEDS1*, *SlEDS5*, and *SlPAD4,* respectively, were upregulated upon infection with *Cmm* (Fig. 3a and Table S3). In Arabidopsis, both of these mutants defected the accumulation of SA [47–49]. The SA levels in tomato cotyledons after infection with *Cmm* showed a similar pattern to the transcripts of defense-associated genes (Fig. 3b). Both *SlPAD4* and *SlEDS1* encoded a lipase-like protein, but their biochemical functions are not yet well-understood. *EDS5* encodes the MATE family transporter and may be responsible for the transport of isochorismic acid from the plastid to cytosol [68]. SA may be formed by either the ICS or PAL pathway [26–29]. Through *Cmm* infection, genes involved in the PAL pathway, including *PAL*, *4Cl*, and *AIM1*, were upregulated, whereas no metabolic enzyme genes of the ICS pathway changed, implying that SA is synthesized through the PAL pathway in tomato (Fig. 3c and Table S3). However, these results are not sufficient evidence for SA synthesis in tomato via the PAL pathway because metabolic intermediates of this pathway are also used as substrates for other compounds, such as polyketide [69]. In Arabidopsis, CBP60s regulate SA synthesis by directly activating ICS1 and PBS3 of the ICS pathway [66, 67]. Whether the relationship between CBP60s and the ICS pathway is conserved in tomato is of interest. Since the accumulation of SA may also be regulated by the posttranslational modification of biosynthetic enzymes or the transport of metabolic intermediates, further detailed studies are required in future. ET, another defense-associated hormone, is formed by ACS and ACO [24]. *LeACO1* increased after infection with *Cmm* (Table S3), which is in accordance with the results of a previously reported microarray analysis [32]. In addition, LeACS2, encoding the rate-limiting enzyme of ET biosynthesis [24], was induced after infection with *Cmm* (Table S3). However, ET only regulates leaf blight symptoms and does not affect bacterial growth in the host leaf. The accumulation of JA was not detected in the tomato cotyledon after infection with *Cmm*, and the expression of the synthetic enzyme genes; LOX2, AOS, AOC, OPR3, and JAR1, was not induced. In summary, the phytohormone controlling the *Cmm* response is suggested to be SA. Previous transcriptomic and proteomic studies have not found any involvement of SA in immunity to *Cmm*. A major contribution to the apparent involvement of SA in response to *Cmm* has been the incorporation of recent hormone results in GO annotation.

The role of SA in response to *Cmm* was examined by the exogenous application of SA to tomato seedlings. SA treatment suppressed the bacterial growth in tomato cotyledons, suggesting that it stimulated immunity to *Cmm* (Fig. 4a, b). The results suggest that SA can be used as a target for the control of *Cmm* in agriculture. However, SA treatment did not completely suppress the growth of bacteria in this study. Because tomato seedlings wilted when treated with SA at concentrations above 2 mM in our experimental system (data not shown), SA is difficult to utilize for bacterial control. The use of plant activator with a priming effect that stimulates SA signaling could be used to control against *Cmm* [70]. qRT-PCR analysis demonstrated that SA treatment induces the expression of WRKY genes (Fig. 4c). These WRKY genes responded to infection with *Cmm* and may be responsible for the expression of defense-associated genes [16]. The suppression of bacterial growth by SA treatment may be due in part to WRKY-mediated immunity. WRKYs may regulate the SA-mediated induction of defense-associated genes after infection with *Cmm*-responsive genes. However, the six PR genes shown in Fig. 2 did not change with SA treatment (data not shown), implying that the defense-associated genes regulated by SA were not entirely consistent with those responding to *Cmm*. The induction of PR gene expression may require other signals derived from *Cmm* in addition to the SA signal. Future transcriptome experiments of SA treatment and the combination of SA and bacterial infection will provide a more detailed understanding of the response to *Cmm* in tomato.

## Conclusions

In this study, transcriptome sequences in tomato cotyledons after *Cmm* infection were analyzed by RNA-seq. Overall, 1788 and 540 genes were identified as upregulated and downregulated DEGs respectively. The expression of defense-associated genes, including PR genes, was induced after infection with *Cmm*, suggesting that plant immunity also functions against Gram-positive bacteria. After infection, many RGAs — including some RLKs responsible for PTI — were transcriptionally upregulated. The expression of *WRKYs*, *NACs*, *HSFs*, and *CBP60s* encoding transcription factors was all upregulated, implying their involvement in defense-associated gene expression during tomato–*Cmm* interactions. After infection with *Cmm*, SA levels increased dramatically, concomitant with the upregulation of genes responsible for SA accumulation like orthologues of Arabidopsis *EDS1*, *EDS5/SID1*, and *PAD4/EDS9*. The application of exogenous SA suppressed bacterial growth and induced the expression of WRKY genes in tomato, indicating that SA plays an important role in the immune response to *Cmm*. Overall, the present study had identified candidate genes involved in *Cmm* infection in PTI, which suggests that SA signaling is a potential target for pest control against *Cmm* in agriculture.

## Materials and methods

### Inoculation of *Cmm* in the tomato cotyledons

Tomato (*Solanum lycopersicum*) cultivar Moneymaker (accession No. TOMJPF00002) was provided by the University of Tsukuba, Tsukuba Plant Innovation Research Center, through the National Bio-Resource Project (NBRP) of the AMED, Tsukuba, Japan. *Cmm* subsp. *michiganensis* virulent strain MAFF301040 was provided by the MAFF GenBank, National Agriculture and Food Research Organization (NARO). Tomato seedlings grown in a chamber under 16 h of light at 25 °C in soil for 10 days were used for inoculation. The bacteria culture of *Cmm* was resuspended at 1 × 10^7^ cfu/mL in infiltration buffer with 10 mM of MES, 10 mM of MgSO_4_, and 0.02% (vol/vol) Silwet L-77. Cotyledons were dipped in the bacterial suspension in closed conical tubes and infiltrated by pressurization with a syringe. After washing the surface of the cotyledons with water, the seedlings were transplanted to soil and cultured under high humidity.

The SA application was performed by transplanting the 10-day-old seedlings into soil moistened with water containing 1 mM of SA and 0.07% (vol/vol) ethanol. Soil moistened with water containing 0.07% (vol/vol) ethanol was used as the control. The effect of SA on resistance to *Cmm* was investigated by inoculating 24 h after transplantation to soil containing SA.

### Determination of the bacterial biomass by qPCR

The bacterial biomass in the plant tissues was quantified by measuring *Cmm* genomic DNA relative to tomato genomic DNA by qPCR analysis. Total DNA was extracted from cotyledons in extraction buffer containing 0.5% (wt/vol) sodium dodecyl sulfate, 25 mM EDTA, 250 mM NaCl, and 200 mM Tris-HCl (pH 8.0) at 60 °C for 30 min, followed by chloroform/isoamyl alcohol (24:1, vol/vol) purification and isopropyl alcohol precipitation. Real-time PCR analysis was performed using the TB Green Premix Ex Taq II (Tli RNase H Plus; TaKaRa Bio, Shiga, Japan) and specific primers for the *Cmm* two-component system sensor kinase gene (ptssk) [71] or plant cytochrome oxidase gene (COX) [72] listed in Table S8. Reactions for real-time PCR were subjected to 40 cycles of 95 °C for 10 s and 60 °C for 1 min using an AriaMX (Agilent Technologies, Palo Alto, CA, USA).

### RNA isolation and RNA-seq analysis

In this study, seven conditions of host transcriptome sequences were analyzed, which consisted of 0 dpi (pre-inoculation), 1, 3, and 6 dpi of mock (buffer) inoculation, and 1, 3, and 6 dpi of *Cmm* inoculation. Each condition included three biological replicates, and a total of 21 samples were analyzed using the Illumina NextSeq500 sequencer. Total RNA was isolated using the RNeasy plant mini kit (Qiagen, Valencia, CA, USA). Library preparation was performed using the SureSelect Strand-Specific RNA Library Prep System (Agilent Technologies). The library was sequenced using the Illumina NextSeq500 system (Illumina, Inc., San Diego, CA, USA) with 75 bp single-read. After trimming, high-quality transcript reads were mapped to the tomato reference genome SL4.0 and ITAG4.0 [73] using CLC Genomics Workbench version 12.0 software (Katrinebjerg, Aarhus N, Denmark).

RNA-seq analysis was performed in triplicate and the TPM value was used as the transcript level. After the log2 transformation of TPM + 1, gene expression levels were compared using one-way analysis of variance (ANOVA) followed by FDR analysis [74]. The correlation coefficient values in samples were calculated from mean TPM values by “cor” function in R. To identify the tomato genes that were responsive to *Cmm* infection, DEG analysis was conducted. In this study, DEGs were identified as the genes with a maximum mean TPM value of 7 treatment areas > 10, a FDR < 0.01 based on a one-way ANOVA, and the average max/min difference being > 2. Upregulated and downregulated DEGs were identified as a fold-change > 2 or < 0.5 when comparing a mean TPM value in mock inoculation conditions with those in the *Cmm* inoculation conditions respectively. For cluster analysis, the Euclidean distances were calculated using z-score transformed data of the TPM value. The phylogenetic tree was constructed by unweighted pair group method with arithmetic mean analysis. Using the “cutree” function in R, upregulated DEGs were classified into the five following clusters: low expression until 3 dpi then greatly increasing at 6 dpi (cluster 1); increased expression at 3 dpi then maintaining a high level until 6 dpi (cluster 2); increased expression at 1 dpi then maintaining a high level until 6 dpi (cluster 3), increased expression until 3 dpi then deceasing at 6 dpi (cluster 4), and increased expression at 1 dpi then decreasing at 3 dpi (cluster 5). The relative gene expressions were visualized with a heatmap.

### Hypergeometric distribution test

Functional annotation of the protein sequences of ITAG4.0 was conducted by DIAMOND searches [75] with a more sensitive mode against UniProtKB (Swiss-Prot + TrEMBL; https://www.uniprot.org). The GO terms were assigned for each of the genes using Blast2GO [76] according to the similarity searches. The candidates of disease RGAs encoding NBSs, RLKs, RLPs, and TM-CCs were searched by RGAugury [19]. The lists of RLK genes were provided by Sakamoto et al. (2012) [22]. The list of TF genes was obtained from the plant TF database PlantTFDB 5.0 [42]. The tomato homologs of CBP60 were identified by BLAST search using Arabidopsis CBP60g as a query. To infer the functional properties of the genes responsive to *Cmm* infection, GO enrichment analyses were performed for the upregulated and downregulated DEGs. Hypergeometric enrichment analysis was performed using the “phyper” function in R. In GO enrichment analysis, *p-*values were adjusted by GO category using FDR analysis with the threshold set to 0.01. In gene set enrichment analysis, gene families with *p*-value < 0.05 were considered to be significant.

### Quantification of transcripts by qRT-PCR analysis

Quantification of gene expression was analyzed by two-step qRT-PCR analysis. First-strand cDNA was synthesized using the PrimeScript RT reagent kit (TaKaRa Bio). Real-time PCR analysis was performed using TB Green Premix Ex Taq II and AriaMX as described above. The primer sequences are listed in Table S8. The expression data were normalized to those of the elongation factor gene *EF1 alpha* [77].

### Measurement of SA

The SA level of the cotyledons was quantified by LC-MS/MS analysis. The cotyledons frozen by liquid nitrogen were ground by Shake Master (Biomedical Science, Tokyo, Japan) and suspended by methanol (200 μL per 100 mg frozen sample) containing 7-hydroxy-5-methylflaone as the internal standard (IS). After centrifugation at 20,000×g for 10 min, the supernatant was collected and the method was repeated once, altering the extraction solvent to 75% methanol with IS. The extract was then filtered using a 0.2 μm-pore hydrophilic PTFE membrane (Millex-LG, Millipore, MA, USA) and the resulting extract was then used for LC-MS/MS analysis.

A LC-MS/MS system consisting of a Nexera X2 liquid chromatograph and a LCMS-8050 triple quadrupole mass spectrometer was used for the quantification of SA with multiple reaction monitoring (MRM) analysis. SA was separated by the InertSustain AQ-C18 column (2.1 × 100 mm; 1.9 μm particle; GL Science, Tokyo, Japan) with multi-step gradient elution of eluents A (water with 0.1% formic acid) and B (acetonitrile with 0.1% formic acid). The gradient elution was performed as follows: 2% eluent B to 50 and 98% in 10 and 15 min, respectively. The column was washed with 98% eluent B for 2.5 min, then re-equilibrated for 2.5 min. The flow-rate and column temperature were kept at 0.4 mL/min and 40 °C, respectively.

An electrospray ionization source was used to detect SA and IS. The source parameters were as follows: nebulizer gas flow, 3 L/min; heating gas flow, 10 L/min; interface temperature, 300 °C; desolvation line temperature, 250 °C; heat block temperature, 400 °C; drying gas flow, 10 L/min. In the MRM experiment, the parameters were optimized using authentic standards as summarized in Table S9. The stability of the overall analysis was evaluated by the coefficient of variance (< 15%) of peak areas of IS.

## Supplementary Information

**Additional file 1: Table S1.** Read counts and mapping status for sequence data.

**Additional file 2: Table S2.** Mean TPM values and correlation coefficient values in samples.

**Additional file 3: Table S3.** List of upregulated DEGs.

**Additional file 4: Table S4.** List of downregulated DEGs.

**Additional file 5: Table S5.** GO enrichment analysis of upregulated and downregulated DEGs.

**Additional file 6: Table S6.** GO enrichment analysis at each time point.

**Additional file 7: Table S7.** Hypergeometric distribution test of TF genes in upregulated DEG.

**Additional file 8: Table S8.** The primers used in this study.

**Additional file 9: Table S9.** Optimized MRM experimental parameters for SA and IS.

**Additional file 10: Figure S1.** Expression of WRKY genes in tomato plants after infection with *Cmm.*

## Data Availability

RNA-seq data were deposited in the DDBJ Sequence Read Archive (DRA) at the DNA Data Bank of Japan (http://trace.ddbj.nig.ac.jp/dra) under the accession number DRA011479 (BioProject; PRJDB111060).

## Abbreviations

*Cmm*: *Clavibacter michiganensis* subsp. *michiganensis*
RGA: Resistance gene analog
RLK: Receptor-like kinase
TF: Transcription factor
SA: Salicylic acid
DEG: Differentially expressed gene
