# Supplementary material for: Transcriptome analysis of Clavibacter michiganensis subsp. michiganensis-infected tomatoes: a role of salicylic acid in the host response
Source: BMC Plant Biol. 2021 Oct 19;21:476. doi: 10.1186/s12870-021-03251-8 (PMC8524973; doi:10.1186/s12870-021-03251-8)
Supplement: Supplementary file 10 — Additional file 10: Figure S1. Expression of WRKY genes in tomato plants after infection with Cmm. [file 12870_2021_3251_MOESM10_ESM.pdf]

Figure S1

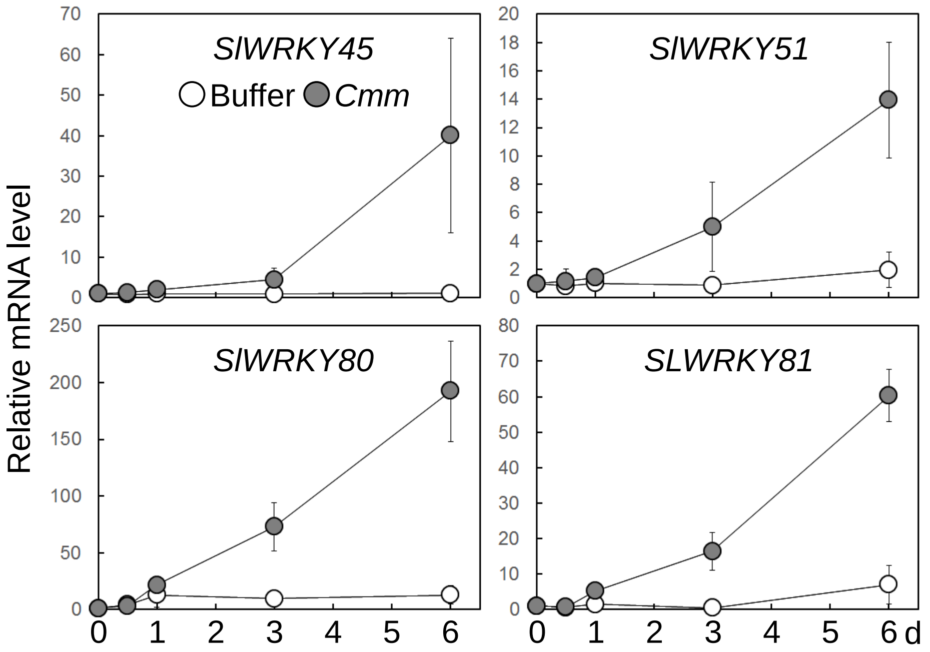

Fig. S1. Expression of WRKY genes in tomato plants after infection with *Cmm*. The transcript levels in tomato cotyledons were quantified by qRT-PCR analysis and expressed relative to the transcript level at 0 day, which was assumed to be one. Data are represented as mean values ± the standard deviation for three replicates.
